# LINAC-based single-isocenter stereotactic radiotherapy for multiple brain metastases

**DOI:** 10.1093/jrr/rrag064

**Published:** 2026-08-12

**Authors:** Hikaru Niitsu, Yoshio Tamaki, Yuichi Hiroshima, Motohiro Murakami, Reiko Kanuma, Toshiyuki Okumura, Yoshinobu Furuyama, Kazuya Shinoda, Hideyuki Sakurai

**Affiliations:** Department of Radiation Oncology, Ibaraki Prefectural Central Hospital, Ibaraki Cancer Center, Kasama, 309-1793, Japan; Department of Radiation Oncology, Faculty of Medicine, University of Tsukuba, Tsukuba, 305-8576, Japan; Department of Radiation Oncology, Ibaraki Prefectural Central Hospital, Ibaraki Cancer Center, Kasama, 309-1793, Japan; Department of Radiation Oncology, Fukushima Rosai Hospital, Iwaki, 973-8403, Japan; Department of Radiation Oncology, Ibaraki Prefectural Central Hospital, Ibaraki Cancer Center, Kasama, 309-1793, Japan; Department of Radiation Oncology, Faculty of Medicine, University of Tsukuba, Tsukuba, 305-8576, Japan; Department of Radiation Oncology, National Cancer Center Hospital East, Chiba, 277-8577, Japan; Department of Radiation Oncology, Ibaraki Prefectural Central Hospital, Ibaraki Cancer Center, Kasama, 309-1793, Japan; Department of Radiation Oncology, Faculty of Medicine, University of Tsukuba, Tsukuba, 305-8576, Japan; Department of Radiation Oncology, Ibaraki Prefectural Central Hospital, Ibaraki Cancer Center, Kasama, 309-1793, Japan; Department of Radiation Oncology, Ibaraki Prefectural Central Hospital, Ibaraki Cancer Center, Kasama, 309-1793, Japan; Department of Radiation Technology, Ibaraki Prefectural Central Hospital, Kasama, 309-1793, Japan; Department of Radiation Technology, Ibaraki Prefectural Central Hospital, Kasama, 309-1793, Japan; Department of Radiation Oncology, Ibaraki Prefectural Central Hospital, Ibaraki Cancer Center, Kasama, 309-1793, Japan; Department of Radiation Oncology, Faculty of Medicine, University of Tsukuba, Tsukuba, 305-8576, Japan

**Keywords:** LINAC-based, single isocenter, stereotactic radiotherapy, multiple brain metastases

## Abstract

Advances in chemotherapy and radiotherapy have expanded indications for stereotactic radiosurgery/radiotherapy (SRS/SRT) for multiple brain metastases. We introduced the Elements Multiple Brain Mets System (mSRS/SRT system), using the dynamic conformal arc method with a single isocenter, for treatment of multiple brain metastases with SRS/SRT (mSRS/SRT). This study analyzed outcomes of mSRS/SRT. From December 2018 to March 2023, 29 patients (130 lesions) were treated using the mSRS/SRT system. The prescribed dose for the planning target volume, determined by D95%, was 21 or 18 Gy in a single fraction for a tumor diameter ≤2 cm and 27 Gy in 3 fractions or 35 Gy in 5 fractions for a tumor diameter >2 cm. Median follow-up time was 7.2 months. The 12-month overall survival rate was 48.5%, and systemic therapy after mSRS/SRT was associated with better overall survival. The 12-month lesion-based local recurrence rate was 14.4%, and larger tumor diameter was associated with poorer local control, whereas systemic therapy after mSRS/SRT was associated with better local control. The 12-month intracranial control rate was 43.6%, and non-lung primary tumors, a greater number of lesions, and systemic therapy before mSRS/SRT were associated with poorer intracranial control. Acute adverse events included 4 cases of Grade 1 headache, one of Grade 1 nausea, and one of Grade 2 alopecia. Brain necrosis was observed in 9 patients (13 lesions), and all cases were asymptomatic. These results suggest that the mSRS/SRT system may become a treatment option for multiple brain metastases, although further evaluation is needed.

## INTRODUCTION

Brain metastases are common occurrences with rates of 10–30% in all patients with cancer [1, 2]. Treatment options include surgery, systemic therapy, radiotherapy and best supportive care, with whole brain radiotherapy as the main method for multiple brain metastases historically. Advances in chemotherapy, particularly molecularly targeted drugs, have improved the prognosis for cases with brain metastasis and extended overall survival (OS) [3]. However, prolonged OS has become a problem with regard to chronic adverse effects such as cognitive function decline due to brain irradiation. To mitigate such adverse events, stereotactic radiosurgery/radiotherapy (SRS/SRT) has been developed to reduce radiation doses to brain tissue without metastases while delivering high doses with high precision to metastatic lesions. However, conventional SRS/SRT typically only allows irradiation of one lesion at a time.

Advances in radiotherapy techniques have expanded indications for SRS/SRT for multiple brain metastases [3–7]. The SRS/SRT platform includes gamma knife, cyber knife and linear accelerators (LINAC). In Japan, LINAC-based SRS/SRT is increasingly used instead of gamma knife due to improved LINAC techniques [8]. Conventional SRS/SRT irradiates each lesion with a multi-isocenter approach, which prolongs treatment time for cases with a higher number of lesions and irradiated brain area due to overlap of irradiation for each target [9, 10]. However, it is now possible to use single-isocenter SRS/SRT for multiple brain metastasis at the same time, which may reduce the brain burden [10].

At our hospital, we introduced the Elements Multiple Brain Mets System (mSRS/SRT system) developed by Brainlab AG (Munich, Germany) in December 2018, using the dynamic conformal arc method with a single isocenter (Fig. 1). This method has proven efficient, facilitating easy verification and quick execution of SRS/SRT for multiple brain metastases. The aim of this study is to analyze the clinical outcomes of patients treated with the mSRS/SRT system for multiple brain metastases. In this manuscript, SRS/SRT using the mSRS/SRT system is referred to as mSRS/SRT.

**Fig. 1:**
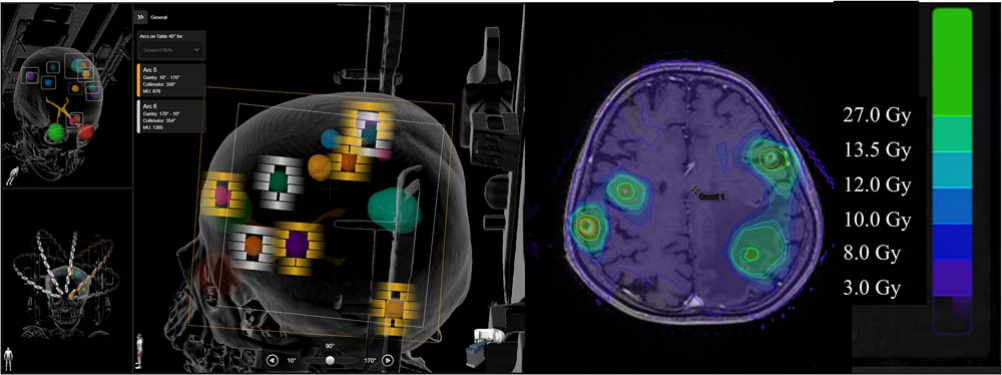
Single-isocenter SRS/SRT using the multiple brain Mets SRS system. Case: 80 years old female. mSRT (27 Gy in 3 fraction) for 12 lesions of brain metastasis from lung cancer.

## MATERIALS AND METHODS

### Patients and study design

At our hospital, patients with multiple brain metastases treated with the mSRS/SRT system between December 2018 and March 2023 were identified from the treatment database. All cases had ≥2 brain metastases. Patients unable to undergo magnetic resonance imaging (MRI) after treatment or for whom follow-up examinations could not be performed were excluded. The cutoff for data was March 2024. Informed consent was obtained in the form of opt-out on a website. The study was approved by the hospital ethics committee (No. 1727).

### SRS/SRT procedure

Patients with multiple brain metastases were treated with the mSRS/SRT system using Varian Trilogy linac with a 5-mm multileaf collimator. Gross tumor volume (GTV) was defined as high signal lesions on Gd-enhanced T1-weighted image. The planning target volume (PTV) included a margin of 2 mm around the GTV. The prescribed dose for the PTV determined by D95% was 21 Gy (or 18 Gy for lesions near the motor cortex or basal ganglia) in a single fraction (fr) for a maximum tumor diameter ≤ 2 cm; and 27 Gy in 3 fr (or 35 Gy in 5 fr for lesions adjacent to an organ at risk such as the brainstem) for a maximum tumor diameter > 2 cm. In cases with maximum tumor diameters of ≤2 and >2 cm, the prescribed dose was selected based on the larger lesion. Dose inhomogeneity within the PTV was limited so that D5% was less than 125% of the prescribed dose. Because this system uses dynamic conformal arc therapy rather than inverse planning such as volumetric modulated arc therapy, beam arrangements and fractionation schedules were selected to satisfy dose constraints for organs at risk.

### Statistical analysis and evaluation of adverse events

OS, local recurrence (LR) and intracranial control rates were evaluated by the Kaplan–Meier method. The rates were defined as the period from the first day of mSRS/SRT to the day of an event. OS and intracranial control were assessed for each patient, and LR was assessed on both a per-lesion and per-patient basis. Patients without an event were censored at the follow-up date. Age, sex, primary site (lung vs. others) and systemic therapy before or after SRS/SRT, Karnofsky performance status, number of lesions, and total tumor volume were evaluated as risk factors.

Adverse events were evaluated with CTCAE v.5.0. An adverse event within 3 months after mSRS/SRT was defined as acute toxicity and a later event was viewed as late toxicity. Brain necrosis was diagnosed based on a T1/T2 mismatch (a lesion with indistinct margins on T2-weighted images, but with well-defined borders on Gd-enhanced T1-weighted images) and elevated apparent diffusion coefficients [11–13]. The cumulative incidence of brain necrosis (cBN) was assessed for each patient, with death treated as a competing event, using the Fine–Gray method. cBN was defined as the period from the first day of mSRS/SRT to the day of an event. Primary site (lung vs. others), fraction number, systemic therapy before or after mSRS/SRT, tumor diameter and total brain volume containing tumor volume irradiated at 12 Gy (*V*_12Gy_) and *V*_24Gy_ were evaluated as risk factors for adverse events. Analysis of *V*_12Gy_ was performed only on mSRS cases and *V*_24Gy_ on mSRT cases.

Associations of risk factors with outcomes and adverse events were analyzed using Cox regression models in uni- and multivariate analysis, with *P* < 0.05 defined as significant. Multivariate analysis was performed on factors with *P* < 0.05 in univariate analysis. IBM SPSS Statistics v.28 (IBM Co., Armonk, NY, USA) was used for statistical analysis.

## RESULTS

### Patient characteristics

During the study period, mSRS/SRT was performed in 39 patients with 163 lesions. Of these, 10 patients with 33 lesions were excluded: 3 patients with 7 lesions were transferred to another hospital soon after treatment and could not be followed up, 5 patients with 21 lesions died within 3 months after irradiation and could not undergo MRI follow-up and 2 patients with 5 lesions received a special dose-fractionation schedule. Consequently, the study included 29 patients with 130 lesions (Table 1). The median age was 66 years (range: 45–84 years). The primary lesions were lung cancer (*n* = 19), colon cancer (*n* = 4), breast cancer (*n* = 3) and other cancers (*n* = 3). The median number of treated lesions was 3 (range: 2–12) and the median maximum tumor diameter was 11.1 mm (range: 5.1–28.8 mm). Treatment used a single fraction in 27 cases (88 lesions) and multiple fractions in 10 cases (42 lesions). For multiple fractions mSRT, 3 fractions was used in 4 cases (22 lesions) and 5 fractions in 6 cases (20 lesions). Repeated mSRS/SRT for recurrence outside the irradiation field was performed in 6 cases. mSRS/SRT was performed twice in 5 cases and four times in 1 case. Prior radiotherapy before mSRS/SRT had been administered in 13 cases, including prophylactic cranial irradiation in 1 case (5 lesions) and SRS/SRT for single brain metastasis in 12 cases (37 lesions). Additional radiotherapy after mSRS/SRT was administered in 14 cases, including whole-brain radiotherapy in 3 cases (8 lesions) and SRS/SRT for single brain metastasis in 11 cases (35 lesions).

**Table 1:** Patient characteristics

Item	Value
Number of cases	29
Sex	Male: 14, Female: 15
Median age (range)	66 (45–84)
Median KPS (range)	80 (30–100)
Primary sites	
Lung	19
Colon	4
Breast	3
Others	3
Number of lesions	130
Median number of treated lesions at a session	3 (2–12)
Median tumor diameter	11.1 (5.1–28.8) mm
Total tumor volume	1.81 (0.24–10.5) cm^3^
mSRS (case/lesion)	27/88
mSRT (case/lesions)	10/42
Repeated mSRS/SRT (cases)	6
Systemic therapy	
Before mSRS/SRT (case/lesions)	19/92
After mSRS/SRT (case/lesions)	22/87
Both before and after mSRS/SRT (case/lesions)	13/56

Abbreviations: KPS, Karnofsky performance status; SRS, stereotactic radiosurgery; SRT, stereotactic radiotherapy; mSRS/SRT, SRS/SRT using the elements multiple brain mets system.

### Efficacy

The median follow-up time was 7.3 months (range: 3.0–52.1 months) in all patients and 21.4 months (range: 4.6–52.1 months) for survivors. The 12-month OS was 48.5% (95% CI: 38.8–58.2%) (Fig. 2). In univariate analysis, primary site (lung vs. others) (*P* = 0.002), number of lesions (*P* = 0.028), total tumor volume (*P* = 0.017) and use of systemic therapy before (*P* = 0.044) and after (*P* < 0.001) mSRS/SRT were significant prognostic factors for OS. In multivariate analysis, systemic therapy after mSRS/SRT was the only significant factor (*P* = 0.034) (Table 2). The 12-month LR rate on a per-lesion basis was 14.4% (95% CI: 10.2–18.6%) and 32.6% (95% CI: 21.1–44.1%) on a per-patient basis (Fig. 3). In univariate analysis for lesion-based LR, tumor diameter (*P* = 0.011), fraction size (*P* = 0.023) and systemic therapy after mSRS/SRT (*P* = 0.016) were significantly related to LR, and tumor diameter (*P* = 0.018) and systemic therapy after mSRS/SRT (*P* = 0.004) remained significant in multivariate analysis. In univariate analysis for patient-based LR, no significant factors were identified (Table 3). The 12-month intracranial control rate was 43.6% (95% CI: 33.6–54.1%) (Fig. 4). In univariate analysis, primary site (*P* = 0.029), number of lesions (*P* = 0.045) and systemic therapy before mSRS/SRT (*P* = 0.035) were significant factors for intracranial control, and all remained significant in multivariate analysis (*P* = 0.029, *P* = 0.034, *P* = 0.048) (Table 4).

**Fig. 2:**
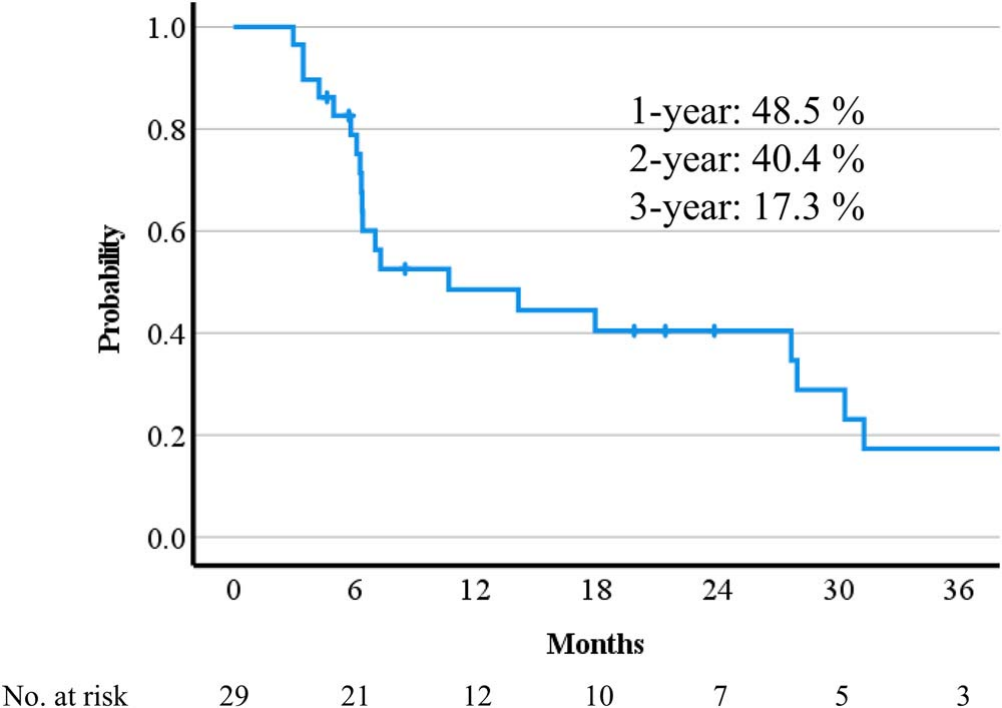
Overall survival (OS) rate.

**Table 2:** Univariate and multivariate analyses of overall survival

Factor	Uni *P*-value	HR (95% CI)	Multi *P*-value	HR (95% CI)
Age	0.50	1.02 (0.96–1.08)		
Sex (female (ref) vs. male)	0.49	0.73 (0.30–1.78)		
KPS	0.84	1.00 (0.97–1.02)		
Primary sites (lung (ref) vs. others)	0.002	4.20 (1.68–10.5)	0.063	2.50 (0.95–6.56)
Number of treated lesions at a session	0.028	1.24 (1.02–1.49)	0.53	1.10 (0.82–1.47)
Total tumor volume	0.017	1.23 (1.02–1.41)	0.087	1.18 (0.98–1.42)
Systemic therapy before mSRS/SRT (no (ref) vs. yes)	0.044	2.93 (1.03–8.35)	0.21	2.18 (0.65–7.25)
Systemic therapy after mSRS/SRT (no (ref) vs. yes)	<0.001	0.15 (0.052–0.44)	0.034	0.29 (0.090–0.91)

Abbreviations: HR, hazard ratio; ref, reference.

**Fig. 3:**
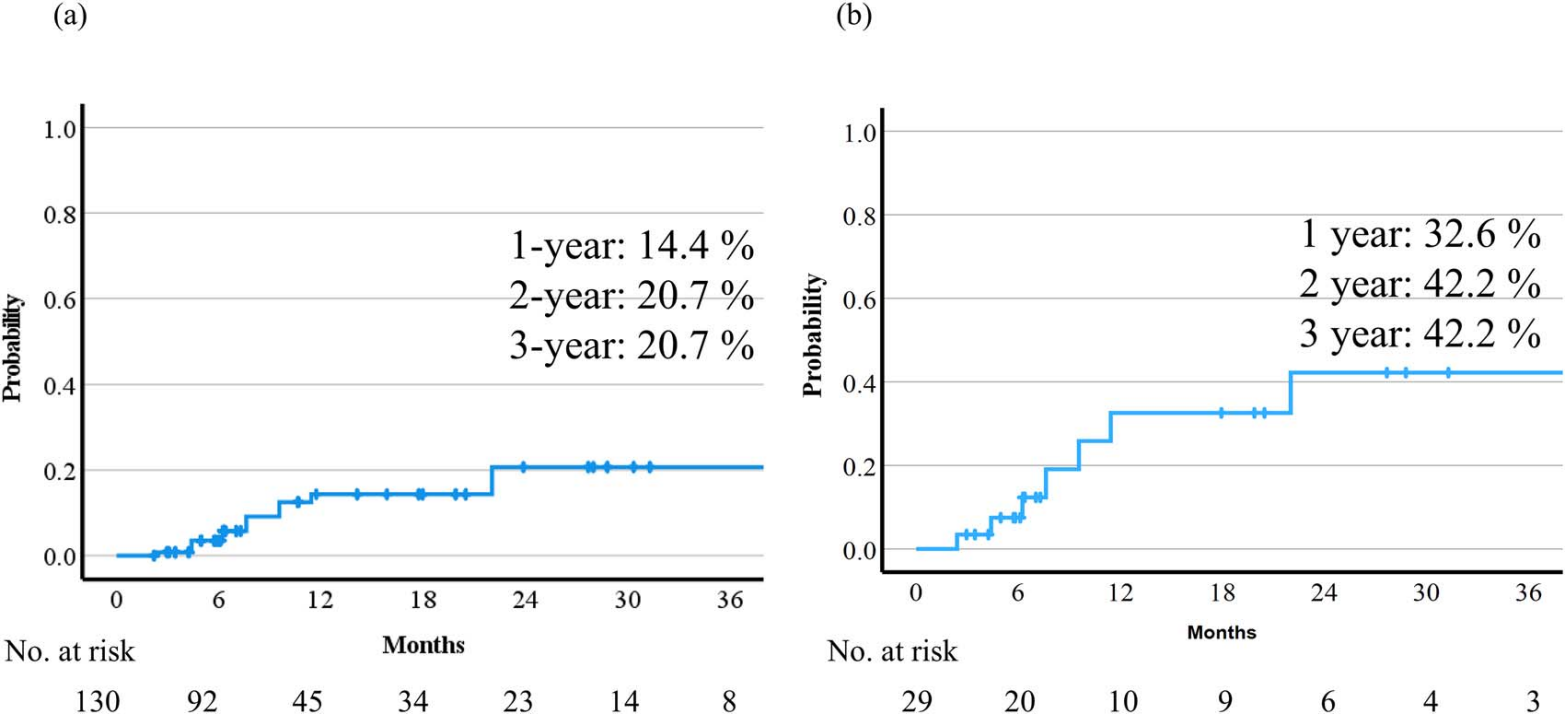
Local recurrence (LR) rate (a) lesion-based and (b) patient-based.

**Table 3:** Univariate and multivariate analyses of local recurrence

Factor	Uni *P*-value	HR (95% CI)	Multi *P*-value	HR (95% CI)
**Lesion-based analysis**				
Primary sites (lung (ref) vs. others)	0.36	0.39 (0.049–3.02)		
Tumor diameter	0.011	1.12 (1.03–1.21)	0.018	1.13 (1.02–1.25)
Fractions (1 fr (ref) vs. multi fr)	0.023	4.04 (1.21–13.5)	0.21	2.34 (0.62–8.84)
Systemic therapy before mSRS/SRT (no (ref) vs. yes)	0.067	4.27 (0.90–20.3)		
Systemic therapy after mSRS/SRT (no (ref) vs. yes)	0.016	0.21 (0.061–0.75)	0.004	0.12 (0.030–0.51)
**Patient-based analysis**				
Primary sites (lung (ref) vs. others)	0.76	0.71 (0.081–6.24)		
Total tumor volume	0.45	1.11 (0.84–1.47)		
Fractions (1 fr (ref) vs. multi fr)	0.086	4.24 (0.81–22.1)		
Systemic therapy before mSRS/SRT (no (ref) vs. yes)	0.053	9.02 (0.97–84.1)		
Systemic therapy after mSRS/SRT (no (ref) vs. yes)	0.079	0.11 (0.009–1.29)		

**Fig. 4:**
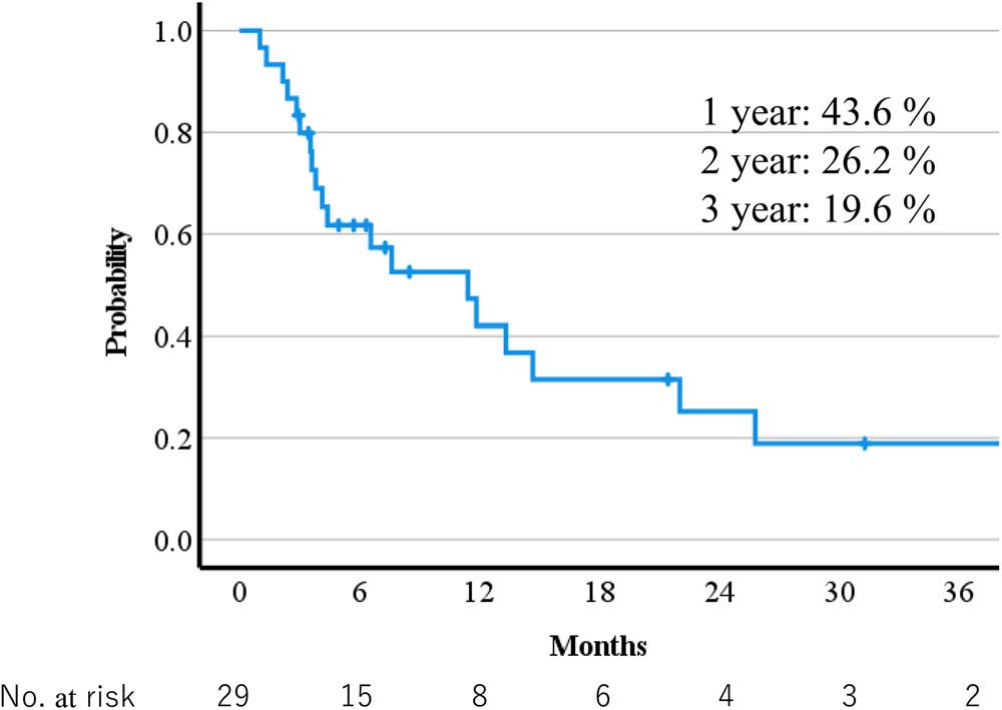
Intracranial control rate.

**Table 4:** Univariate and multivariate analyses of intracranial control rate

Factor	Uni *P*-value	HR (95% CI)	Multi *P*-value	HR (95% CI)
Age	0.63	1.01 (0.96–1.07)		
Sex (female (ref) vs. male)	0.99	1.00 (0.39–2.54)		
KPS	0.14	1.03 (0.99–1.06)		
Primary sites (lung (ref) vs. others)	0.029	3.08 (1.12–8.46)	0.029	3.23 (1.13–9.24)
Number of treated lesions at a session	0.045	1.24 (1.01–1.54)	0.034	1.28 (1.02–1.61)
Total tumor volume	0.051	1.17 (1.00–1.38)		
Systemic therapy before mSRS/SRT (no (ref) vs. yes)	0.035	3.27 (1.09–9.81)	0.048	3.16 (1.01–9.89)
Systemic therapy after mSRS/SRT (no (ref) vs. yes)	0.081	0.32 (0.088–1.15)		

### Toxicity

Acute adverse events included 5 cases of Grade 1 headache, 1 of Grade 1 nausea and 1 of Grade 2 alopecia. Brain necrosis was seen in 13 lesions (10.0%) in 9 patients (31.0%). All cases were asymptomatic, and no patients required treatment with steroids or bevacizumab. 5case (7 lesions) were treated with mSRS, and 4 cases (6 lesions) were treated with 5 fraction mSRT. The 12-month cBN rate was 24.1% (95% CI: 10.4–41.0%). No significant risk factors for brain necrosis, including the presence or absence of radiotherapy before or after mSRS/SRT, were identified in the univariate analysis (Fig. 5 and Table 5).

**Fig. 5:**
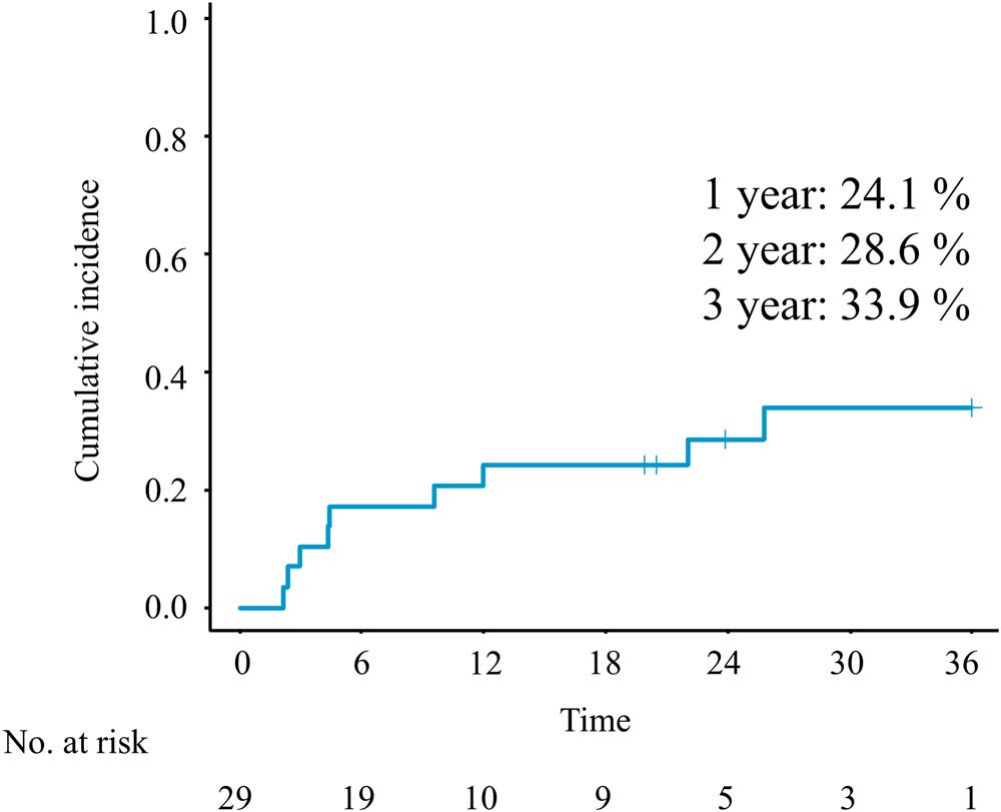
Cumulative rate of brain necrosis(cBN).

**Table 5:** Univariate analysis of cumulative brain necrosis

Factor	*P*-value	HR (95% CI)
Primary sites (lung (ref) vs. others)	0.47	0.54 (0.11–2.82)
Total Tumor volume	0.80	0.98 (0.82–1.16)
Fractions (1 fr (ref) vs. multi fr)	0.32	1.95 (0.53–7.13)
Systemic therapy before mSRS/SRT (no (ref) vs. yes)	0.21	0.45 (0.13–1.56)
Systemic therapy after mSRS/SRT (no (ref) vs. yes)	0.39	2.63 (0.29–23.6)
*V*_12Gy_ for mSRS	0.38	0.97 (0.91–1.04)
*V*_24Gy_ for mSRT	0.55	0.97 (0.88–1.07)
Radiation therapy before or after mSRS/SRT (no (ref) vs. yes)	0.47	0.62 (0.17–2.23)

Abbreviations: *V*_12Gy_ and *V*_24Gy_, tissue volume containing the tumor volume irradiated at 12 Gy and 24 Gy.

## DISCUSSION

Single-isocenter mSRS/SRT system has been considered a promising treatment option for patients with multiple brain metastases. Compared to conventional multi-isocenter SRS/SRT, this method allows dose concentration without changing the brain dose [14] and reduces the incidence of brain necrosis [15] with no difference in LR [16]. However, rotational errors are more likely as the distance from the isocenter increases, which may reduce dose concentration to the tumor [17], but the risk of LR or necrosis has been found to be unchanged [18, 19]. These results suggest that SRS/SRT using a single isocenter may be an equivalent treatment to conventional multi-isocenter SRS/SRT [20].

Studies of treatment outcomes for single-isocenter SRS/SRT for multiple brain metastases are limited and heterogeneous. Reports utilizing volumetric modulated arc therapy have shown a 1-year OS rate of 53.5%, a LR rate of 1.0–13.3%, and an intracranial control rate of 31.4–40% [21–23]. In the first report of single-isocenter dynamic conformal arc SRS for up to 10 lesions, the 1-year OS and LR rates were 68% and 11%, respectively [24]. Another study using the same treatment system as that at our center for patients with 4 to 10 lesions had a median OS of 10.4 months and intracranial progression-free survival of 7.1 months [25]. More recently, a prospective registry study of single-isocenter dynamic conformal arc SRS for multiple brain metastases has also been reported, further supporting the clinical feasibility of this approach [26]. In the present study, the 1-year OS was 48.5%, the LR rate was 14.4% and the intracranial control rate was 43.6%, which appeared to be within the range reported in previous studies. However, these findings should be interpreted with caution because of the small sample size and the presence of selection bias.

Reported prognostic factors for outcomes after single-isocenter SRS/SRT include age, sex, primary tumor site, number of brain metastases and tumor margin dose for OS [21, 27]; age, primary tumor site, number of metastases and peripheral dose for intracranial recurrence [27] and total tumor volume [21] and total dose [28, 29] for LR. Use of systemic therapy has also been associated with OS, PFS [30] and LR [31]. In our cohort, systemic therapy after mSRS/SRT was associated with better OS. In the lesion-based analysis of LR, larger tumor diameter was associated with poorer local control, whereas systemic therapy after mSRS/SRT was associated with better local control. No significant factors were identified in the patient-based analysis of LR. For intracranial control, non-lung primary tumors, a greater number of lesions and systemic therapy before mSRS/SRT were associated with poorer outcomes. Interestingly, systemic therapy before and after mSRS/SRT showed opposite trends in our analyses. Although molecular targeted agents and immune checkpoint inhibitors have been reported to improve treatment outcomes [31], systemic therapy before mSRS/SRT may have reflected a subgroup with more advanced or treatment-resistant disease. In contrast, patients who were able to receive systemic therapy after mSRS/SRT may have had better preserved general condition or disease control, which may partly explain their more favorable outcomes. Similar tendencies were also noted in a previous report [30]. Univariate analysis showed significant associations with several of the previously reported factors, but these relationships were not retained in multivariate analysis. This may be due to the relatively small sample size and low incidence of LR, and also to confounding factors and multicollinearity among clinical variables. The limited variability in treatment parameters and biological heterogeneity among primary tumor types may also have influenced the statistical data. Furthermore, patients with short follow-up periods or those who died early were excluded from the primary analysis, and these associations may therefore have been influenced by substantial selection bias. Therefore, findings regarding efficacy should be interpreted with caution. These factors should be considered when interpreting the findings and in design of studies in larger, more diverse patient populations.

The rates of acute adverse events with single-isocenter SRS/SRT have been reported to be 1.4–6.2%, 0–8% and 0% for Grades 2, 3 and ≥4, respectively [10, 21, 32]. In this study, the Grade 2 rate of 1/29 cases (3.4%) was similar. Late adverse events, particularly brain necrosis, of Grade ≥2 have ranged from 1.4% to 4.6% in previous studies [10, 21, 32], whereas all cases with brain necrosis in our cohort were Grade 1. Although the patient-based cumulative incidence of brain necrosis in our cohort appeared higher than that reported in previous studies, all cases were Grade 1, with no severe events observed. In addition, the small sample size and the presence of repeated stereotactic irradiation before or after mSRS/SRT in some patients may have influenced the estimated incidence. Therefore, these findings should be interpreted cautiously. The risk of brain necrosis is proportional to *V*_12Gy_ in SRS [7, 33]. For SRT, multiple factors have been reported depending on the fractionation schedule: *V*_20Gy_ and *V*_24Gy_ for 3-fraction and *V*_24Gy_ and *V*_30Gy_ for 5-fraction [7, 33]. In the Fine–Gray analysis, fraction size showed a trend toward an association with brain necrosis, although no clear differences were observed in *V*_12Gy_ or *V*_24Gy_. The past report investigating differences in brain necrosis between SRS and SRT indicate a trend of lower rates with SRT, but there were no significant differences [34]. In this study, brain necrosis was frequently observed in the mSRT group, particularly at 5-fraction mSRT. Among the four necrosis cases in the 5 fr-mSRT group, three patients had undergone SRT for one recurrent lesion before or after mSRS/SRT. In contrast, none of the five necrosis cases in the SRS group had a history of stereotactic irradiation before or after mSRS/SRT. These findings suggest that additional stereotactic irradiation before or after mSRS/SRT may have affected brain dose. This method uses dynamic conformal arc therapy, raising concerns about the difficulty in adjusting brain dose. However, no clear statistical association was identified between the history of radiotherapy before or after mSRS/SRT and brain necrosis in this cohort. Given the small number of events, the possible influence of repeated stereotactic irradiation cannot be excluded. This suggests the need to allocate single or multiple fractions taking into account both tumor size and LC and the risk of brain necrosis. Because brain necrosis often develops relatively late after stereotactic irradiation, its incidence may have been underestimated in patients with short follow-up. Likewise, the assessment of local control may have been influenced by the inclusion of patients with limited follow-up. In addition, because patients with very short follow-up or early death were excluded from the main analysis, substantial selection bias may also have affected the estimated incidence of brain necrosis. Guidelines recommend follow-up every 2–3 months for the first 2 years post-treatment, followed by every 4–6 months thereafter [3]. However, the diagnostic accuracy of Gd-enhanced MRI may be insufficient [35], and it is also unclear if a greater MRI frequency improves diagnostic accuracy. Adding other evaluations using diffusion-weighted imaging or positron emission tomography may enhance accuracy, but no standard method has been established [13]. Therefore, within the scope of current methods, follow-up MRI after treatment should be performed whenever possible to distinguish necrosis from recurrence.

There are some limitations in this study, including its retrospective observational design in a small number of cases, which raises a concern of selection bias. In addition, patients without post-treatment MRI evaluation or follow-up were excluded, including those who died before follow-up, which may have introduced further selection bias and affected the assessment of treatment efficacy and adverse events, particularly OS. Because patients with very short follow-up or early death were not included in the main analysis, treatment efficacy may have been overestimated and the incidence of adverse events may also have been affected. There was also a bias in the distribution of primary tumor sites, and due to this difference, systemic therapy before and after SRS/SRT differed, which may have affected treatment outcomes. There were also differences in the dose fractionation.

Given the limitations of this study, further evaluation of the mSRS/SRT system in a larger number of cases is needed. Nevertheless, the present results suggest that the mSRS/SRT system has the potential to become one of the treatment options for multiple brain metastases.

## CONFERENCE PRESENTATION

The 35th Annual Meeting of Japanese Society for Radiation Oncology, The 36th Annual Meeting of Japan Conformal External Beam Radiotherapy Group/JASTRO, ESTRO meets ASIA 2024.

## CLINICAL TRIAL REGISTRATION NUMBER

1727
